# Diphenhydramine Intoxication With Blood Extended Half-Life and a False Positive Result for Tricyclic Antidepressants

**DOI:** 10.7759/cureus.63540

**Published:** 2024-06-30

**Authors:** Koji Yokoyama, Asuka Kaizaki-Mitsumoto, Satoshi Numazawa, Mitsukazu Mamada

**Affiliations:** 1 Department of Pediatrics, Japanese Red Cross Wakayama Medical Center, Wakayama, JPN; 2 Department of Toxicology, Showa University Graduate School of Pharmacy, Tokyo, JPN

**Keywords:** systemic toxicity, false-positive, blood concentration measurement, tricyclic antidepressants, diphenhydramine overdose

## Abstract

Diphenhydramine is a first-generation antihistamine medication. Acute intoxication with diphenhydramine can be severe and potentially fatal. The current case is of a 13-year-old girl who presented with central nervous system depression after voluntary intake of unknown drugs. Serum concentration analysis showed diphenhydramine intoxication, blood half-life extension, and a false positive result for tricyclic antidepressants (TCAs) in urine examination. To our knowledge, this is the first reported case of confirmed diphenhydramine overdose with a false positive result for TCAs and measurement of the serum level in a child. Considering the similarities between the clinical symptoms of diphenhydramine and TCA intoxication, this case illustrates that all physicians should consider the possibility of cross-reactivity during the diagnosis of patients with unknown acute drug intoxication who test positive for TCAs.

## Introduction

Diphenhydramine is a first-generation antihistamine used for symptoms of allergies and colds. The symptoms caused by excessive intake of diphenhydramine and its prognosis vary, but overdose of this agent was responsible for 3.2% of excessive drug use-related deaths in the U.S. in 2016 [1,2]. The diphenhydramine half-life period is shorter in children than in adults and elderly patients [3]. Tricyclic antidepressants (TCAs), increasingly prescribed for multiple indications in children and adults, are responsible for many pediatric poisonings [4]. Among adolescents who overdosed on drugs with suicide intent, 2.63% took TCAs [5]. For TCA overdose, there were 1.12 exposures per 10,000 population [6]. In 2022, it was reported that 460 people aged 13-19 years in the U.S. overdosed on TCAs [7]. In clinical settings, TCA screening assays have historically had a high rate of false positives [8]. Moreover, the similarities between the clinical symptoms of diphenhydramine and TCA intoxication mean that medical doctors may misdiagnose patients and administer the wrong treatment [9].

## Case presentation

**Figure 1:**
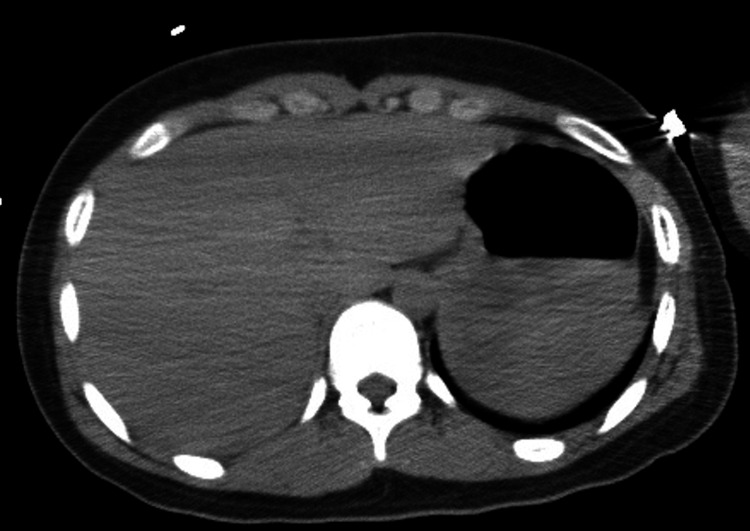
Abdominal computed tomography examination Low absorption areas found in the stomach

A 13-year-old girl was admitted to our emergency department due to babbling, irritability, and myoclonic movements of the limbs after voluntary intake of unknown drugs. Her past and familial histories were unremarkable. Her initial vital signs were blood pressure of 134/74 mmHg, heart rate of 140 beats/minute, respiratory rate of 20 breaths/minute, and temperature of 37.7 degrees. Her Glasgow Coma Scale score was 9 (E3V2M4). Her pupils were 4 mm bilaterally and properly reacted to light. No signs of injury were found on her body. Brain computed tomography examination revealed no abnormality. She was uncooperative and we could not obtain accurate information about the ingested drugs. The results of laboratory tests including complete blood count, biochemical test, coagulation test, urine test, blood gas test, chest radiography test, and electrocardiogram test were normal. Therefore, to identify the causative drug, a urine screening test for drugs and their metabolites was performed using a commercial kit (Signify ER^TM^; Abbott Diagnostics Medical Co., Ltd., USA) [10]. No test indicated significant intoxication of any drug except for TCAs. This product can screen for 11 substances of abuse and TCAs had a sensitivity of 60.0%, specificity of 92.4%, false positive of 4.0%, and false negative of 40.0% (from the manufacturer information). The product with the same effect as Signify ER^TM^ in Japan was iCASSETTE™ Dx in the U.S. [11]. Within 30 minutes after visiting the emergency service, abdominal computed tomography examination revealed low-density areas, indicative of a drug mass within her stomach (Figure 1). We initially diagnosed her with TCA intoxication and admitted her to the intensive care unit for gastric irrigation, charcoal administration, hydration, cardiorespiratory monitoring (including non-invasive blood pressure measurement, continuous pulse, oxygen saturation, and urine volume measurement, and regular blood gas analysis), and close physical observation. Over the next 24 hours, her consciousness gradually improved. From her mother, we learned that she had simultaneously ingested 2700 mg of diphenhydramine (18 capsules and 36 tablets, 50 mg/capsule and tablet). After regaining consciousness, the patient confessed she had bought drugs at two local pharmacies to commit suicide and denied orally ingesting TCAs. After her physical condition stabilized, her mental state remained stable. She calmly stated that no clear factors led to her suicide attempt and that she thought she would feel better after taking this drug. We performed tricyclic and tetracyclic antidepressant screening tests using a serum sample before treatment, and no test indicated the presence of these agents. The patient fully recovered without specific intervention at 36 hours after admission. She had a good neurological outcome and was discharged from the hospital with outpatient psychiatry follow-up. Her serum concentration of diphenhydramine was 1.39 μg/mL at the time of arrival and 0.129 μg/mL at 42.25 hours after admission. This concentration was measured by gas chromatography-mass spectrometry as previously described [12]. We diagnosed her with diphenhydramine intoxication with a false positive result for TCAs.

## Discussion

The course of this patient raises two important clinical points. First, ingestion of a large amount of diphenhydramine can cause a false positive result for TCAs in urine. We confirmed that the patient had overdosed on diphenhydramine and not taken TCAs by performing blood concentration measurements. Second, the apparent half-life wound delay in diphenhydramine overdose in the patient. Six hours of follow-up is generally required [13]. However, longer follow-ups may be required. Antihistamine mediators, specifically diphenhydramine and cyproheptadine, carbamazepine (anticonvulsant), and cyclobenzaprine (commonly used muscle relaxant), cause false positive results in TCA screening. These substances all possess ringed structures, which simulate the tricyclic rings in some toxicity screens [14]. Drugs that cause false positives have similar structural formulas to TCAs, and these similarities cause the observed interferences [8]. Among cases who had taken large amounts of diphenhydramine, some false positive results for TCAs have been reported [15]. The exact mechanism by which diphenhydramine intoxication results in a false positive result for TCAs is unclear [15]. Diphenhydramine is lipophilic and has a large volume of distribution. Serum levels of diphenhydramine peak 2-3 hours after oral administration. The elimination half-life of diphenhydramine varies between age groups; it is approximately five hours (range: 4-7 hours), nine hours (range: 7-12 hours), and 13.5 hours (range: 9-18 hours) in pediatric, adult, and elderly patients, respectively [3]. Blood concentration analysis of our patient showed that the drug remained in her body, despite infusion therapy, gastric lavage, and activated charcoal administration. The apparent half-life would be about 12.3 hours. Physicians should pay attention to diphenhydramine toxication for a longer period. This is a single case report, and further case studies are required for clinical applications. 

## Conclusions

This case illustrates that all physicians should consider the possibility of cross-reactivity during the diagnosis of patients with unknown acute drug intoxication who test positive for TCAs in rapid testing and should consider the appropriate observation time for patients who have overdosed on diphenhydramine to commit suicide, especially in pediatric cases.
